# Study of the Air-Entraining Behavior Based on the Interactions between Cement Particles and Selected Cationic, Anionic and Nonionic Surfactants

**DOI:** 10.3390/ma13163514

**Published:** 2020-08-09

**Authors:** Qi Liu, Zhitao Chen, Yingzi Yang

**Affiliations:** 1 School of Civil Engineering, Harbin Institute of Technology, Harbin 150090, China; soulworld67@gmail.com (Q.L.); rainczt@hotmail.com (Z.C.); 2 Key Lab of Structures Dynamic Behavior and Control of the Ministry of Education, Harbin Institute of Technology, Harbin 150090, China; 3 Key Lab of Smart Prevention and Mitigation of Civil Engineering Disasters of the Ministry of Industry and Information Technology, Harbin Institute of Technology, Harbin 150090, China

**Keywords:** AEA, surfactant, air void system, foam index, zeta potential, ATR-FTIR spectroscopy

## Abstract

The essential role of the air void size distribution in air-entrained cementitious materials is widely accepted. However, how the air-entraining behavior is affected by features such as the molecular structure of air-entraining agents (AEAs), the type of solid particles, or the chemical environment of the pore solution in fresh mortars is still not well understood. Besides, methods to assess the interaction between AEAs and cement particles are limited. Thus, in this study, the air-entraining behaviors of three kinds of surfactant (cationic, anionic, and nonionic) were examined. The general working mechanisms of these surfactants were studied by zeta potential and attenuated total reflectance-Fourier transform infrared (ATR-FTIR) spectroscopy. Results indicate that the cationic surfactant entrains improper coarse air voids due to the strong electrical interaction between air bubbles formed by the cationic surfactant and negatively charged cement particles. The anionic surfactant interacts with the positively charged part of cement particles, and thus entrains finer air voids. The interaction between the nonionic surfactant and cement particles is very weak; as a result, the nonionic surfactant entrains the finest and homogeneous air voids.

## 1. Introduction

An air-entraining agent (AEA) is a kind of chemical admixture that can entrain air voids throughout concrete by mechanical mixing. Tiny and well-dispersed entrained air voids can promote the freeze-thaw durability of concrete by releasing the pressures during freezing and thawing cycles, or improve the workability of concrete by the lubrication effect [1,2,3,4,5,6]. Although considerable attention has been focused on measurement and characterization of the air content in concrete, the actual air void size distribution is the most critical parameter when AEA is used, whether to better offer frost resistance or obtain proper rheology [3,7,8]. Thus, a more in-depth research is needed to reveal the mechanisms of AEA’s influence on the air void system.

Primarily, AEAs are surfactants that contain hydrophilic and hydrophobic moieties [9,10,11]. Depending upon the charge on the hydrophilic headgroup of the surfactant molecule, AEAs can be divided into four groups: anionic, cationic, nonionic, and amphoteric [12,13]. Most AEAs used in field concrete are anionic types, such as lignin sulfonate and sulfonated hydrocarbon-soluble salts, and a few are nonionic [14]. Rare records have been reported about the use of cationic surfactants as AEAs [15], and thus more information is needed for a systematic comparison among these AEAs.

**Figure 1:**
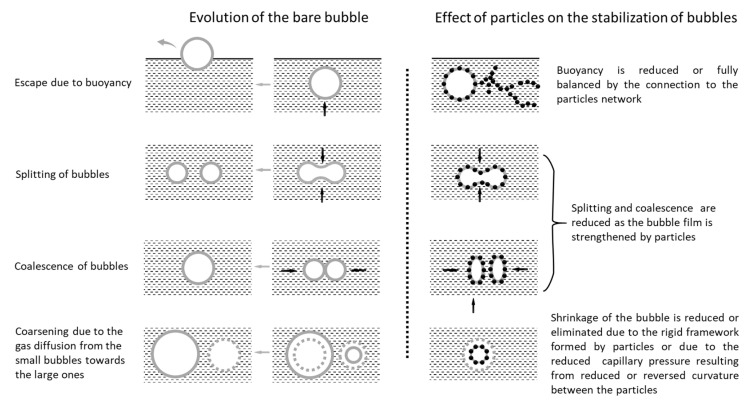
Schematic of the effects of particles on the stabilization of bubbles.

When an AEA is added to a cementitious material, a strong interaction between air bubbles and solid particles occurs [16,17,18], which could be the origin of the air-entraining behavior. It has been widely accepted that the attachment of particles to the air bubble surface may improve the bubble stability [19,20]. The adsorption of particles onto an air bubble decreases its buoyancy and may connect the air bubble to the particle network in the bulk aqueous solution [19], thus keeping the air bubble suspended in the aqueous phase and preventing the air bubble from splitting or coalescing [21]. At the same time, the coarsening effect, which may reduce the volume of small bubbles or fully dissolve small bubbles due to gas diffusion between bubbles, is highly reduced by the attachment of particles [21,22,23]. These effects of particles on the stabilization of bubbles are summarized and illustrated in Figure 1.

Although there have been extensive works dealing with the air-entraining performance, there is a paucity of information on the interaction between solid particles and surfactants due to the limitations of test methods such as surface tension or foam height measurements [24,25,26]. Thus, test methods involving solid phases are needed. In some studies, the zeta potential is used to study the interaction between surfactants and cement particles [27,28,29,30]. Results show that the zeta potential behavior can reflect the adsorption mechanism of surfactants onto solid particles. Meanwhile, in situ attenuated total reflection-Fourier transform infrared (ATR-FTIR) spectroscopy was introduced in a recent study of a cementitious system as a surface-sensitive technique that can provide mechanistic information on adsorption [31,32,33,34]. Via spectroscopic study, the structure–property relationship of the surfactant and cement phase is revealed, and some adsorption models can be established.

There have also been some reports on the adsorption behavior of superplasticizers and cement; so far, the literature shows a limited number of studies dealing with the interaction between AEAs and cement particles, and further, the associated air-entraining behavior. In this study, the most critical questions are: how does the electrical property of the surfactant head group impact the interaction between surfactant and cement, and how does this influence the air-entraining behavior? Therefore, the present work aims to ascertain the effect of anionic, cationic, and nonionic surfactants on the entrained air void system. The air-entraining behavior was first tested by the air void size distribution of hardened mortars and the foam index performance. Then, the zeta potentials of cement particles in surfactant solutions were determined to reveal the possible mechanisms influencing the air-entraining behavior. Furthermore, in situ ATR-FTIR spectra were measured to study these mechanisms.

## 2. Materials and Methods

### 2.1. Air-Entrained Mortar Mixtures

Portland cement (P.I 42.5) complying with GB175-2007 [35] and provided by Dalian Cement Group Co., Ltd (Dalian, China). was used in the test. China ISO standard sand produced by Xiamen ISO Standard Sand Co. Ltd. was used as fine aggregate. For the preparation of mortars, a classical structural mortar mix proportion with a water–cement–aggregate ratio of 1:2:6 (by weight) was adopted. 

**Figure 2:**
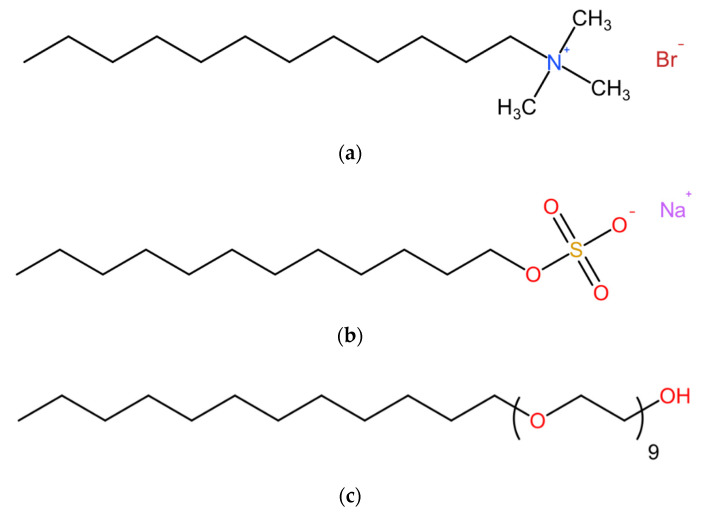
Schematic diagram of the molecular structure of the surfactants used in this work. (**a**) Dodecyltrimethyl-ammonium bromide (DTAB). (**b**) Sodium dodecyl sulfate (SDS). (**c**) Fatty alcohol polyoxyethylene ether-9 (AEO-9).

Three representative surfactants with alkyl tails of the same length were used in this research: a cationic surfactant (dodecyltrimethyl-ammonium bromide, DTAB), a commercial anionic AEA based on sodium dodecyl sulfate (SDS), and a nonionic surfactant (fatty alcohol polyoxyethylene ether-9, AEO-9). The molecular structures of each surfactant are exhibited in Figure 2.

For each mortar sample, the AEA was first added to the mixing water, after which the binder and sand were added. The mortar was mixed in a cement mortar mixer at a low speed of 140 ± 5 rpm for 90 s followed by a high-speed mixing of 285 ± 10 rpm for 120 s. The air content of the fresh mortar was measured after the cement was in contact with water for 5 min. The concentration of surfactant in the cement mortars to achieve 10% air content was 80, 80, and 75 mg/L of water for the cationic (DTAB), anionic (SDS), and nonionic (AEO-9) surfactants, respectively.

### 2.2. Air Void Parameters of Hardened Mortar at 28 d

After 28 d of water-curing, approximately 100 × 100 × 20 mm^3^ samples were cut from cube mortars that had air contents between 9% and 10% in a fresh state, then were ground flat and polished. The sampling procedure generally followed the guide of Ref. [36]. The tested surface was polished with successively finer grit sizes at first until the edges of air voids as small as 10 µm were sharp and distinguishable. The polished surfaces were sprayed with water-soluble black ink. After the ink dried, 50 nm titanium dioxide powder was pressed into the air voids. Then, the excess powder was carefully scraped off by the edge of a stainless steel blade to obtain a clear sample surface in a black background with white air voids. Sections of each sample with dimensions 80 × 80 mm^2^ were photographed using an opto-digital microscope (OLYMPUS DSX500, OLYMPUS Corporation, Tokyo, Japan). The resolution of the captured images was about 3200 dpi, corresponding to an 8 μm pixel size. The air void size distribution was calculated by Image-Pro Plus 6.0 (Media Cybernetics, Silver Spring, MD, USA).

### 2.3. Foam Index Test

**Figure 3:**
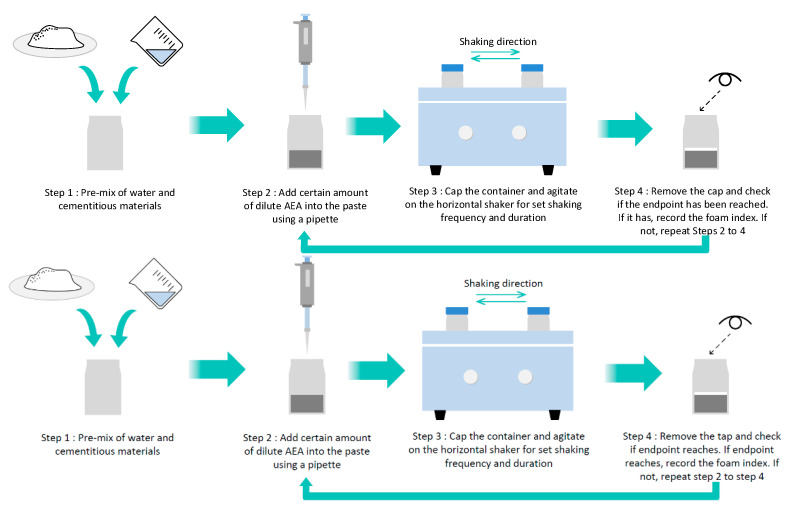
Schematic diagram of the foam index test procedure.

The foam index (FI) test was used to determine the foaming behavior of the AEAs [37]. This method involves titrating the AEA into a dilute cement paste, followed by vigorous agitation. When a stable layer of foam can be formed on the top of the paste, the endpoint is reached, and the amount of AEA required is defined as the foam index [38]. In this study, for each FI test, 22-mm-thick cement slurry with or without fly ash with a water-to-cement ratio of 2 was pre-mixed in a glass bottle, and then a known volume of AEA solution was added. Then, the slurry was agitated by a horizontal shaker for 120 s, after which the slurry surface was visually monitored. If a stable foam layer at the water–air interface was observed to remain stable for at least 45 s, the endpoint was reached and the quantity of AEA was recorded as the foam index. If a stable foam layer was absent, another drop of the AEA solution was added and the above process was repeated. In this research, fly ash provided by Shuangda Power Station (Harbin, China) was adopted, and depending on the adsorption capacity of the solid phases, the AEA was diluted 10 to 200 times to limit the cycle to about 20 times. The general testing procedure is shown in Figure 3. For detailed study of the foam index test method, please refer to [38].

### 2.4. Zeta Potential Measurements

The zeta potential of cement particles was measured by a Malvern Zetasizer Nano ZS (Malvern Instruments Ltd., Malvern, UK) at 20 °C. The instrument measures the electrophoretic mobility of particles in dilute suspension in a controlled electric field. Then, the zeta potential is calculated by utilizing the Smoluchowski equation [39]. The cement particles were ground before the tests to meet the test requirements. Then, around 0.02 g of cement and 20 mL of solution (with or without surfactant) were mixed by hand-shaking for about 15 s followed by ultrasonic treatment for 2.5 min. Subsequently, 1 mL of suspension was transferred into the measuring cell for zeta potential measurement. After 2 min of temperature equilibrium, the zeta potential was measured. Three runs were conducted for each sample, with the average value being the final result. 

### 2.5. In Situ Attenuated Total Reflection-Fourier Transform Infrared Spectroscopy 

Infrared spectra were obtained in a reflective absorbance mode (ATR-FTIR) with a Nicolet iS10 FT-IR (Thermo Scientific Instrument, Waltham, MA, USA) spectrophotometer equipped with a diamond crystal. Measurements were performed at 1 cm^−1^ resolution in the range of 650–1800 cm^−1^ at room temperature. For each ATR-FTIR measurement, a 30 μL surfactant solution droplet was first pipetted onto the diamond crystal to cover the crystal surface fully, and was scanned as background. Then, approximately 0.01 g cement was carefully added to this droplet. Later, a spectrum was acquired. Similar to the method used in Ref. [40,41,42], the resulting spectrum was obtained by subtracting the surfactant solution from the spectrum of surfactant–interface–cement; therefore, the recorded spectrum was expected to show the bands of the cement, as well as the result of interactions between the surfactant solution and cement particles in the interfacial region. When surfactant molecules are adsorbed from the solution to the cement particle surfaces, the packing structure or chemical environment of the surfactant molecules may change, resulting in specific features of the spectrum. Since the shape and position of the peaks between the spectra obtained after the cement particles were in contact with water for 1 to 15 min were not significantly different, only the spectra at 5 min are illustrated. 

## 3. Results and Discussion

### 3.1. Air Void Structure of Hardened Mortars

**Figure 4:**
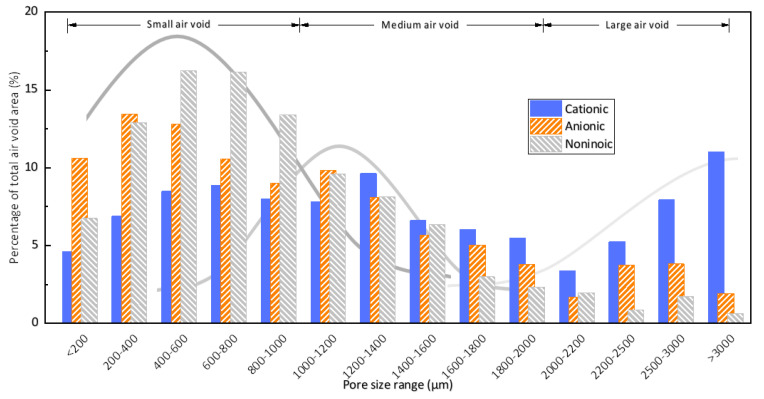
Air void size distribution of hardened mortars with different surfactants.

The air void size distributions of mortars with different kinds of surfactant are shown in Figure 4. In general, the trends are similar to the findings of other scholars. However, the air voids in this research are coarser than those in concrete due to a lack of coarse aggregate [43,44]. 

As shown in Figure 4, there are two or three peaks of the distribution within the measured range of air void size. The first peak of the air void size distribution of all mix proportion appears in the range of 0 to 1000 μm, and the distribution approximately fits a normal distribution. The second peak roughly lies between 1000 and 2000 μm, and the distribution is not clear, as this peak may overlap with the distribution of the other peaks. The third peak appears in the range above 2000 μm. In this range, the distribution of air void size is less ordered, as the entrainment of larger air voids is entirely random and can be influenced by multiple mechanisms. The appearance of multiple peaks can be observed in many studies, indicating that several mechanisms govern the air-entraining process [7,45,46,47].

Although the air content of each sample was the same, there were great differences in the air void distribution among these samples. For easier comparison, the air content of small air voids, medium air voids, and large air voids was calculated and is illustrated in Table 1. It can be found that for the sample with the cationic surfactant there were many more large air voids, as these air voids took up about 27.56% of the total air voids and the small air voids occupied only 36.88%, resulting in low and flat peaks of medium and small air voids. Meanwhile, for the anionic surfactant, the proportion of large air voids was lesser compared with that observed with the cationic surfactant, resulting in a higher proportion of small air voids (about 56.47%). Meanwhile, for the nonionic surfactant, the amount of large air voids was minimal. The amount of small air voids was very high, about 65.43%, and the peak of the medium air voids was unclear, which means that the air voids were finer and more homogeneous. In summary, the cationic surfactant entrained many large air voids, the anionic surfactant mainly entrained small air voids, and the nonionic surfactant entrained more small air voids. The air void size distributions of different kinds of surfactants suggest that the nonionic surfactant is more suitable for frost protection than the anionic surfactant, and the cationic surfactant is least appropriate for this use.

### 3.2. Foam Index Performance

**Figure 5:**
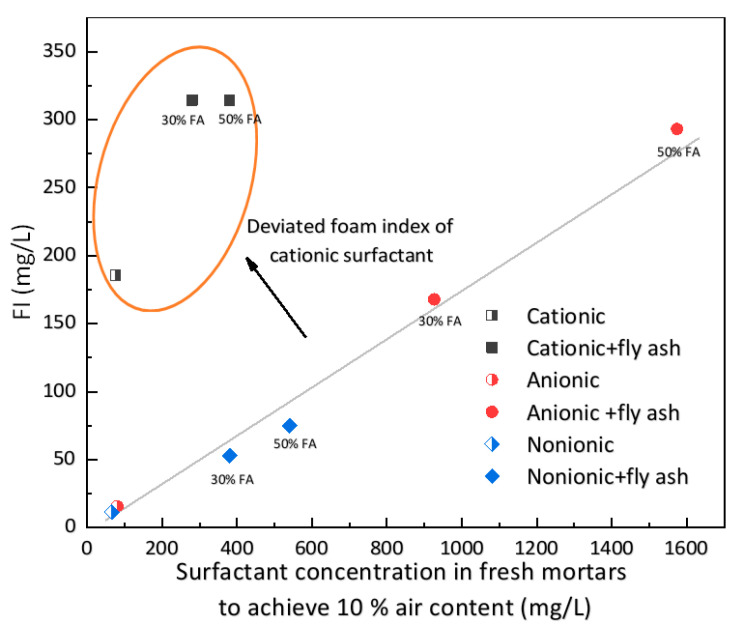
Influence of the type of surfactant on foam index (FI) and surfactant concentrations in fresh mortars to achieve 10% air content with or without fly ash.

Figure 5 presents the influence of the type of surfactant on the FI as well as the surfactant concentrations required in fresh mortars in order to achieve 10% air content with or without fly ash. The performance of anionic and nonionic surfactants generally exhibits a linear correlation between surfactant concentration in the cement mortar and foam index within a wide range of fly ash replacement ratios, as discussed in previous research [38]. However, the use of a cationic surfactant resulted in a larger foam index, as the data points deviate substantially from the predicting line. At the same time, the assessment of endpoint was strongly interfered with, resulting in the apparent failure of the surfactant concentration prediction as the foam index was not changed with increasing fly ash replacement ratio, as shown in Figure 5. Thus, the results of foam index tests reflect the improper air voids entrained by the cationic surfactant.

**Figure 6:**
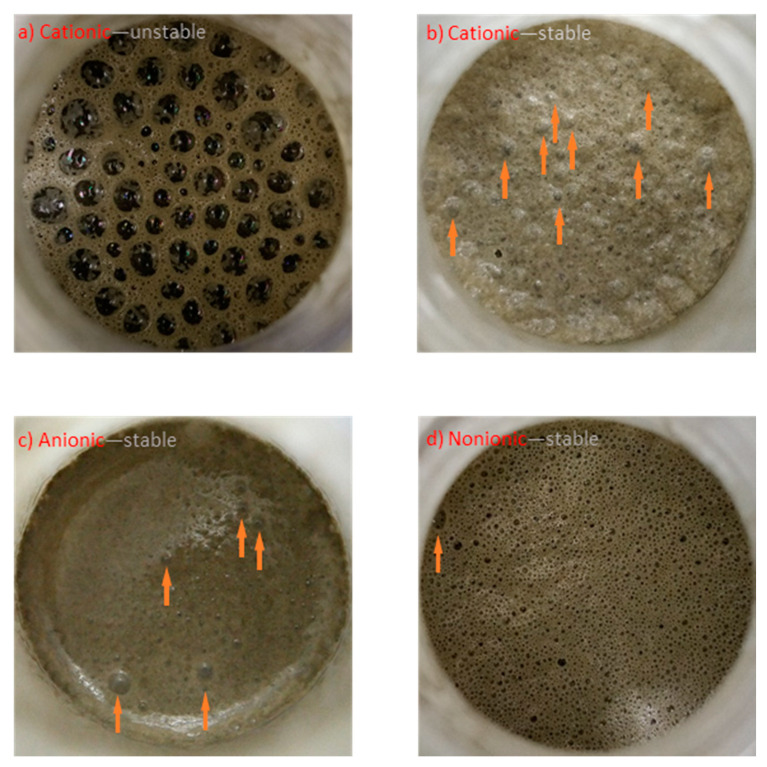
Pictures of foam layers in foam index tests without fly ash. (**a**) Unstable foam of the cationic surfactant; (**b**) stable foam of the cationic surfactant; (**c**) stable foam of the anionic surfactant; (**d**) stable foam of the nonionic surfactant. Arrows show the position of large air bubbles when the endpoint is reached.

The pictures of the foam layer in the foam index tests in Figure 6 indicate that the overestimated cationic surfactant doses in the foam index tests were caused by the overall coarser air voids entrained by the cationic surfactant. As shown in Figure 6a, before the endpoint was reached, the cationic surfactant generated extra-large air bubbles compared to the anionic surfactant (Figure 6c) and the nonionic surfactant (Figure 6d). These extra-large air bubbles could remain stable for several seconds. However, due to the larger size of the air bubbles, their surfaces could only be partially covered by cement particles. Thus, their stability could not be maintained for long enough. The breakup of one extra-large air bubble would cause a local shock followed by a series of successive breakups of nearby air bubbles, resulting in problems with the judgment of the endpoint. Thus, in order to produce a stable foam layer, additional surfactant is needed to reach the endpoint, resulting in a larger foam index. In this circumstance, the air bubbles in Figure 6b are finer than those in Figure 6a, forming a thicker layer of foam. The deviating foam index of the cationic surfactants again indicates that this kind of surfactant may be inappropriate for air entrainment in mortars. 

In addition, the interaction between surfactants and cement particles was different for different surfactant types. When the endpoint was reached, the surfaces of air bubbles with cationic and anionic surfactants were fully covered by cement particles (Figure 6b,c). However, air bubbles formed by the nonionic surfactant were less covered by cement particles, and more dark areas can be observed in Figure 6d because the air or paste can be seen through these air bubbles. The adhesion of cement particles to air bubbles is critical to the stability of the air bubbles, as it strengthens the air bubbles and reduces the breakup of large air bubbles into smaller bubbles. As a result, there are more large air bubbles in Figure 6b,c compared to Figure 6d, as pointed out by arrows. Moreover, the amount of large air bubbles in the foam index tests also follows the order of the content of large air voids in the hardened mortars in Table 1. The cationic surfactant mainly entrained large air bubbles, the anionic surfactant entrained finer air bubbles, and the nonionic surfactant entrained the finest air bubbles. This indicates that the interaction between air bubbles and surfactants plays a significant role in the final air void distribution in cementitious materials.

This assumption can be supported by direct observation of air bubbles raised to the top surface of fresh cement paste [18,48], as in these studies, only the air bubbles formed by AEAs were covered by a dense shell of solid particles and the surfaces of air bubbles without AEA were very clean. However, the air entrainment mechanism of different surfactants cannot be conclusively determined with macroscopic experiments alone. Thus, zeta potential and infrared spectra features were examined, in the hope of providing further mechanistic information on the interaction between cement particles and surfactants, and further, the air entrainment behavior.

### 3.3. Interaction between Cement Particles and Surfactants

#### 3.3.1. Zeta Potential of Cement Particles in Model Solutions

The zeta potential of cement particles in filtered pore solution from the fresh paste was measured first. As the ion concentration in the real pore solution of cement paste was higher than the limitation of the testing instrument (Malvern Zetasizer Nano) and could not be controlled accurately, the recorded zeta potential fluctuated a great deal in the range between −5 and −7 mV. Thus, in further measurements of zeta potential, a model solution was used to offer a proper chemical environment instead of a real pore solution. The model solution consisted of 20 mmol/L Ca(OH)_2_ and 50 mmol/L K_2_SO_4_, which has also been employed by other researchers [49,50,51]. In this model solution, the zeta potential of cement particles was a small negative value of about −5 mV.

#### 3.3.2. Zeta Potential of Cement Particles in Surfactant Solutions

**Figure 7:**
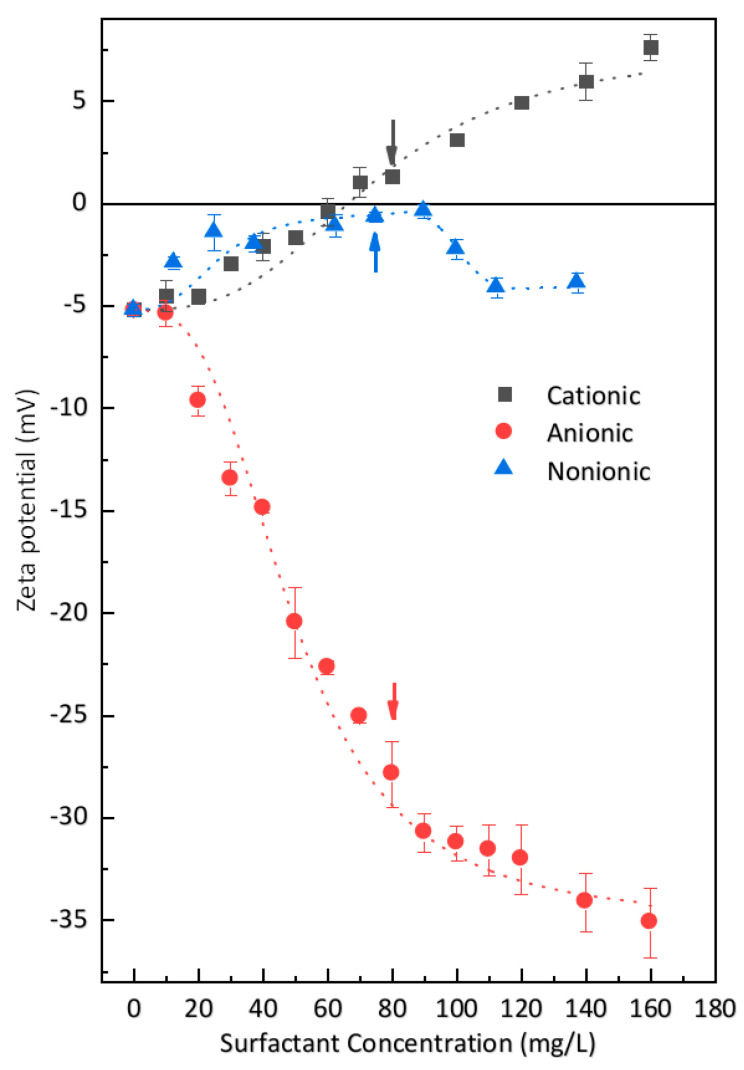
Changes in zeta potential of cement particles with increasing surfactant concentration. Arrows signify the concentration of each surfactant required to reach 10% air content in fresh mortars. Dotted lines are guides for the eyes.

The variation in zeta potential as a function of surfactant concentration in the model solution was measured in order to provide further information about the interaction between cement particles and surfactant molecules. As depicted in Figure 7, the zeta potential of cement particles could be changed by all three kinds of surfactant. This behavior agrees with the research of Zhang et al. [30], as they found that cationic and anionic surfactants could both be adsorbed by cement particles. 

For the cationic surfactant, the zeta potential of cement particles was significantly increased by increasing the amount of cationic surfactant, and the increasing rate did not change very much within the testing concentration range. This means that cement particles could strongly adsorb the cationic surfactant. As the zeta potential of cement particles is negative, the adsorption of the cationic surfactant occurred due to the expected electrostatic interactions.

For the anionic surfactant, the zeta potential was significantly decreased by increasing the amount of surfactant. When above the concentration of 80 mg/L, the rate of decrease was lowered. The decreased zeta potential by the anionic surfactant indicates that anionic surfactant could also be strongly adsorbed by cement particles, which is somewhat confusing since the zeta potential of cement particles is negative. According to the results of Zhang et al. [30], the anionic surfactants also have their hydrocarbon chain towards the liquid phase. This signifies that the negatively charged head group of the anionic surfactant is attached to the negatively charged cement particle surface. Two possible explanations are proposed for this behavior: (i) the anionic surfactant molecule can be adsorbed on the cement particle surfaces by the bridging effect of cations [29] or by the formation of complexes with polyvalent metal cations such as Ca^2+^; (ii) a cement particle is a mixture of multi-mineral phases, and several hydration products with different electrical properties can form on its surface. As a result, the charge distribution of cement particles is heterogeneous [28,52,53,54]. Thus, the anionic surfactant can be adsorbed by some phases with a positive charge, and vice versa.

For the nonionic surfactant, the zeta potential was moderately changed by increasing the surfactant concentration. Within the testing concentration, there was a maximum around the level of 90 mg/L, which is very close to the critical micelle concentration of the nonionic surfactant. Below the maximum value, the zeta potential increased with increasing surfactant concentration, while beyond the maximum value, the zeta potential decreased to a stable value with increasing surfactant concentration. Similar features have been previously noted [55], suggesting hemi-micelle adsorption of the nonionic surfactant onto cement particles [56]. Though this surfactant is nonionic, the ether oxygen of the nonionic surfactant can be considered as a Lewis base, resulting in interaction with SiO_2_ [57]. Thus, several simultaneous weak effects may explain the adsorption behavior [58]: (i) an equilibrium is established by the cooperation of amphoteric hydroxylic groups with a proton; (ii) a complex forms by the combination of surfactant molecules with hydroxylic surface groups; (iii) there is a lateral interaction of hydrocarbon chains between surfactant molecules. When neutral polymers are adsorbed on the surface of solid particles, the zeta potential may be explained by two mechanisms: (i) pushing the slipping plane away from the particle surface, which may reduce the absolute value of the zeta potential; and/or (ii) influencing the adsorption process of ions in the solution [39]. Thus, the variable zeta potential of cement particles by the nonionic surfactant indicates that a limited interaction exists.

### 3.4. Interaction between Cement Particles and Air Bubbles

#### 3.4.1. Model of the Interaction between Cement Particles and Air Bubbles

**Figure 8:**
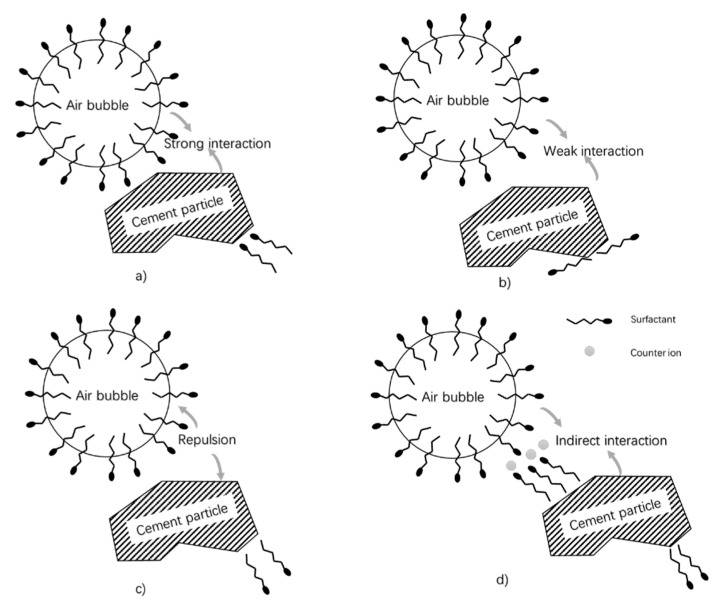
Schematic illustration of the influence of the surfactant’s orientation on the interaction between air bubble and solid particle: (**a**) strong interaction; (**b**) weak interaction; (**c**) repulsion; and (**d**) indirect interaction.

The zeta potential data above demonstrate the presence of an interaction between cement particles and surfactant molecules. However, the interaction of cement particles with surfactant is not equal to that with air bubbles because the attachment of surfactant to cement particles is oriented. As shown in Figure 8, for monolayer adsorption, as in cementitious material the concentration of surfactant is much lower than the critical micelle concentration, the adsorption of surfactant molecules on cement particles may result in different behavior of the adherence of cement particles onto air bubbles. For surfactants adsorbed on cement particles orienting their hydrophobic tails towards the aqueous phase, cement is attractive to air bubbles (Figure 8a). On the contrary, if surfactants are adsorbed on solid particles by hydrophobic adsorption, the hydrophilic groups may orient towards the aqueous phase, resulting in repulsion (Figure 8c) or a much weaker indirect attraction (Figure 8d). If the surfactant molecule is lying parallel to the surface, the interaction between air bubbles and solid particles could be weak (Figure 8b). Thus, more information is needed to explain the adsorption mechanisms. 

#### 3.4.2. ATR Difference Spectra of Cement Particles in Surfactant Solutions

**Figure 9:**
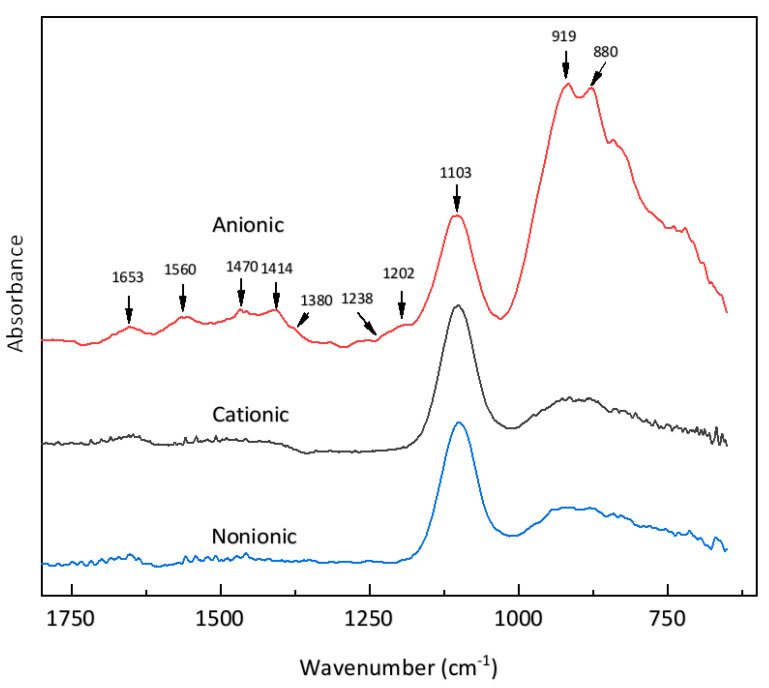
ATR-FTIR difference spectra of surfactants after 5 min of adsorption to cement particles.

Spectroscopy studies provide direct information about the adsorption process and possible structures formed during the adsorption. Thus, they can be used to characterize the orientation of surfactant molecules when adsorbed on cement particles, and to clarify which kind of situation in the model shown in Figure 8 is appropriate for the interaction between cement particles and air bubbles. Figure 9 displays the infrared difference spectra of typical results of adding cement to surfactant solutions. Here, surfactant concentrations of 10 wt% (much higher than in the air-entrained mortars) were used to give more definite results.

For the nonionic surfactant AEO-9, few peaks could be distinguished. Peaks in the 1150–550 cm^−1^ region are mainly the absorption bands of the clinker phases [59]. The band at 1653 cm^−1^ comes from interfacial solvent water [60,61,62]. The broad and small peaks between 1500 and 1400 cm^−1^ arise from various bending modes of the surfactant tail at 1468 cm^−1^ (δ CH_2_), 1420 cm^−1^ (δ α-CH_2_), and 1380 cm^−1^ (δ CH_3_-R) [63]. This featureless peak indicates that changes in the chemical environment of the AEO-9 molecules are not strong enough to form intense peaks.

For the cationic surfactant (DTAB), the spectrum is similar. As the two bands of the asymmetric deformation modes of CH_3_-N^+^ in the DTAB headgroup also appear at 1490 and 1480 cm^−1^ [63], the band around 1400 cm^−1^ is a little higher, which may indicate a stronger interaction with charged sites on the cement particle surface [64]. However, no more notable peaks can be distinguished, indicating that electrostatic interaction causes no intense change of the chemical environment of the cationic surfactant.

Meanwhile, for the spectrum of the anionic surfactant (SDS), more distinct peaks can be observed. The adsorption band near 1476 cm^−1^ is assigned to the -CH_2_ bending (or scissor) mode. The shoulder band near 1380 cm^−1^ is attributed to a -CH_3_ deformation [65]. The CH_2_ scissoring group is susceptible to intermolecular interaction [66], and the enhanced height of this band is also indicative of a decreased activity of interchain coupling [67,68]. The peaks at 1560 and 1410 cm^−1^ can be ascribed to the asymmetric and symmetric stretching of the carboxylate ion (COO-) [69,70,71,72], which may result from the impurity of the commercial air-entraining agent.

The two bands at 1202 cm^−1^ and 1238 cm^−1^ arise from the A mode of ν_as_ S-O and ν_s_ S-O and the E mode of ν_as_ S-O of the headgroup of SDS, respectively [73,74,75]. The A vibration of the SDS molecule at −1200 cm^−1^ is parallel to the surfactant molecule and is sensitive to direct contact with the charged surface or head-to-head adsorption with a cationic surfactant. However, the E vibrational mode at −1240 cm^−1^ is in the direction normal to the surfactant molecule and is more sensitive to lateral interactions between surfactant molecules [73,76,77]. Therefore, the change of these bands results from a difference in the local environment of the sulfonate head during adsorption at the cement particle surface [78,79]. The absorption at ~1200 cm^−1^ occurs when SDS is adsorbed via the OSO_3_^-^ group [74]. Simultaneously, a small loss in intensity for the band at −1240 cm^−1^ will occur upon adsorption [75]. Therefore, according to [73], a promoted band at −1200 cm^−1^ and a depressed band at −1240 cm^−1^ indicate that the sulfate head group interacts directly with positively charged surfaces.

For cement particles, though the net charge is negative, the zeta potentials of different clinker phases and hydrate phases scatter broadly with various solvents. Mainly, the zeta potentials of C_3_S, gypsum, and C-S-H are positive, and the zeta potentials of C_2_S, CH, and ettringite are negative [80,81]. Meanwhile, after a few seconds to several minutes of cement hydration, very fine hydration products are formed, such as ettringite (100–500 nm) and C-S-H (<50 nm) [81,82,83,84]. The deposition of these hydration products on the clinker surface forms very unevenly charged surfaces. Thus, the bands at −1200 cm^−1^ and at −1240 cm^−1^ in Figure 9 are an indication of direct interaction of the anionic surfactant headgroup with the positively charged part of the cement particle. As a result, we can conclude that anionic surfactant molecules orient their tails towards the aqueous phase, as illustrated in Figure 8a. That is to say, a strong interaction between positively charged parts of cement particles and air bubbles was formed in the presence of the anionic surfactant.

### 3.5. Summary

On the whole, our results indicate that the interactions between cement particles and surfactants are dominated by electrostatic interactions. Cationic surfactants can be adsorbed by the negatively charged part of cement particle and anionic surfactant by the positively charged part, orienting their tails to the aqueous phase. The interaction between nonionic surfactant and cement particles is very weak. The interaction between cement particles and surfactant molecules leads directly to an interaction between cement particles and air bubbles, which plays a significant role in the air-entraining behavior [17,85]. When solid particles are attached to air bubbles, these air bubbles are strengthened. At the same time, the velocity of air bubbles is decreased so that larger air voids can be kept in the paste. Due to the net negative zeta potential of the cement particle, the interaction between cement particles and different kinds of surfactants followed the order: nonionic (AEO-9) < anionic (SDS) < cationic (DTAB). Correspondingly, the cationic surfactant entrained coarser air voids. In contrast, the anionic surfactant entrained more fine air voids, and the nonionic surfactant could entrain an even finer air void system, as depicted in Figure 4 and Table 1.

## 4. Conclusions

In this study, the air void size distributions of hardened cement mortars using selected cationic (DTAB), anionic (SDS), and nonionic (AEO-9) surfactants were examined. Foam index tests were performed with three surfactants to illustrate the foaming behavior of these surfactants. The zeta potential was measured to discuss the distinct air-entraining behavior of these surfactants, which results from the interaction between cement particles and surfactants. The possible mechanism of the adsorption of surfactant on the cement particle surface was analyzed by in situ ATR-FTIR. The following conclusions can be drawn:

(1) The selected cationic (DTAB), anionic (SDS), and nonionic (AEO-9) surfactants in this research could all entrain an adequate air content in cement mortars. However, for mortars with about 10% air content, air voids entrained by the cationic surfactant were much coarser, as more than 25% of the entrained air voids were larger than 2000 μm, while the anionic surfactant (SDS) and the nonionic surfactant (AEO-9) entrained mid-sized and smaller air voids, respectively.
(2) Overestimated surfactant dosage in foam index tests is an indication of improper use as an air-entraining admixture in cementitious materials. When the cationic surfactant (DTAB) was used as the air-entraining admixture, it had an overestimated foam index and entrained excessive coarse air voids.
(3) Zeta potential is a method to reflect the interaction between surfactant and cement particles. Due to the overall negative charge of cement particles, the cationic (DTAB), anionic (SDS), and nonionic (AEO-9) surfactants interacted with cement particles differently, according to their electrostatic interactions. Results indicate that the adsorption of the cationic surfactant (DTAB) arises from electrostatic interactions with the negative charge sites of cement particles. Correspondingly, the anionic surfactant (SDS) interacts with the positive charge sites of cement particles. Both surfactant molecules orient their tail to the aqueous phase. The nonionic surfactant (AEO-9) interacted moderately with cement particles. The degree of interaction between cement particles and the selected surfactants followed the order: nonionic (AEO-9) < anionic (SDS) < cationic (DTAB).
(4) Under the condition of monolayer adsorption of surfactants on cement particles orienting their hydrophobic tails towards the aqueous phase, the stronger interaction between surfactant molecules and cement particles results in a stronger interaction between air bubbles and cement particles, which further influences the air void size distribution of hardened mortars. The degree of interaction between cement particles and air bubbles formed by selected surfactants in this study followed the order: nonionic (AEO-9) < anionic (SDS)< cationic (DTAB). The stronger the interaction between air bubbles and the cement particles, the larger the air voids entrained.

The results of this study provide new insights into the air-entraining behavior of different surfactants. Zeta potential measurements of the cement particles in various chemical environments may offer information connecting the air void system and the surfactant molecule structure. In this research, the improper coarse air voids entrained by the cationic surfactant were ascribed to the strong electrical interaction between cement particles and air bubbles due to the negative charge of the cement particles. Thus, investigation of the electrical property of the cementitious system may be helpful in assessing the air entrainment of different surfactants. However, the present study was only conducted with a limited number of surfactants, only cement was used as the tested solid phase, and the model of the interaction between cement particles and air voids was simple. Therefore, more in-depth research involving more surfactants, cementitious materials, and mix proportions should be performed for a clearer characterization of the air-entraining behavior, and a detailed model of the interaction between solid particles and surfactants could be established.

## Figures and Tables

**Table 1:** Summarization of the effect of the type of surfactants on the air void system.

Type of Surfactant	Proportion of Air Voids in Each Distribution Range/%		
	Small Air Voids (0–1000 μm)	Medium Air Voids (1000–2000 μm)	Large Air Voids (2000–3000 μm)
Cationic	36.88	35.56	27.56
Anionic	56.47	32.41	11.12
Nonionic	65.43	29.38	5.19
